# Hydrocele in a Female

**Published:** 1875-02

**Authors:** G. A. Baxter

**Affiliations:** Chattanooga, Tennessee


### HYDROCELE IN A FEMALE.

BY G. A. BAXTER, M.D., CHATTANOOGA, TENNESSEE.

In re-reading Dr. Thomas' work on "Diseases of Women," I find the following paragraph in relation to hydrocele: "This affection, which consists in a collection of fluid in the inguinal canal, around the round ligament, is one of such rarity in the female that its very existence is commonly ignored, and mention of it is rarely made by systematic writers;" and followed by a full report of a case by Dr. E. P. Bennett, of Danbury, Connecticut.

I report, from a sense of duty, the following case, which came under my notice, and the only one that I have heard of among my professional acquaintances.

Mary G——, married, thirty-two years of age, gave the following history of her difficulty on consulting me: Two years previously, when lifting a heavy bucket of coal, she felt a sharp pain in the inguinal region, but it passed away in a few moments, and she thought nothing more of it for a day or two, when she again felt a pain "like a dead heat" in the inguinal region, and extending down the labia majora. There was some swelling, which led her a day or two after to apply to a physician, who gave her a lotion. She used this for a few days with no apparent effect, until, in lying down one day, most of the swelling suddenly disappeared and the pain ceased. She told her Doctor, who "seemed somewhat surprised and chan ed her medicine," but she ceased applying this as useless.

The tumor remained in this same condition until about two weeks previous to her visit to me. At that time, she says she fell over a chair and bruised the labia majora. There was no acute pain, however, but the tumor began to increase in size, and had been doing so up to her visit. On careful examination, I found a tumor large as an egg, with its apex pointing to the external inguinal ring, and its ba=e large and bulging out the upper portion of the labia majora. I concluded at once it was a hernia, and attempted a reduction, but failed. Then, after a more careful examination, I was satisfied I had erred in diagnosis. The tumor gave a slight sense of fluctuation, but was very tense and tender, and I made up my mind that it was an abscess, and without more ado proceeded to open it, never for a moment suspecting hydrocele, or allowing this to enter into my differentiation.

To my surprise, I found a serous fluid, instead of pus, enveloped in a sack. With my scalpel-handle and finger-nail I scraped the cavity, and succeeded in getting a speedy recovery.

